# Aiding Nature

**Published:** 1907-10

**Authors:** 


### AIDING NATURE.

There is a tremenduous lot of talk about aiding nature, mostly sheer rot. It generally amounts to standing by and doing nothing, avoiding active intervention and letting the man and the disease fight it out for themselves.

What do we ever do in our treatment of disease beyond helping nature? Drugs don't act in a dead body, consequently they act through their power of inciting some part of the living body. Let us consider how we may most effectively aid nature, and not rashly and ignorantly interfering embarrass her instead.

No sick person can be the better for having in the bowels a mass of dead, decaying, microbe-infected organic matter, the most deadly in its effects on the human organism of any poison known. No person but must be the worse for the presence of this material, especially at a time when by the presence of fever there is an increased absorption of the fluid portion. We may then confidently say that we aid nature by clearing out the bowels, disinfecting them if necessary, and keeping them clear and clean throughout the attack. By this we clear the way for the digestion, absorption and assimilation of foods, and thus materially aid nature in supporting the patient through the crisis.

Next, we investigate the eliminant apparatus, and note if all are in full activity and able to take care of the increased requirements. Since we recognize the immense importance of toxemia in all infectious maladies, the elimination of these toxins becomes a vital necessity. A man dies if elimination is checked, even without any outside morbific influence. We must eliminate to continue to live. Open the dcors and let out the destructive elements that are poisoning the vital centers, and we aid nature.

The studies of Juergensen opened the eye of the profession to the enormous strain under which the heart labors during an acute attack of pneumonia. To a certain extent this is true of all fevers, and as a general principle of therapeutics it is our duty to see that the heart is not overtasked, and that at the least evidence of weakness we sustain its forces. Assuredly we thus aid nature.

When the vital forces are depressed by any noxious influence we aid nature by removing or opposing these influences that tend toward death. If the patient is being poisoned by emanations from noxious material in or about his premises, we attend to the hygiene and remove such deleterious elements—and we have repeatedly urged on the profession our conviction that herein lies the true source of malignity in infectious maladies. On the same principle we seek to destroy the living germs of infectious disease by saturating our patient with sulphides, and to reinforce the leucocytes by administering nuclein solution, in both cases believing we are directly and effectively aiding nature.

Whenever we note a relatively weak part in the body, or a correspondingly virulent attack on any part, as denoted by the symptoms, we seek to strengthen that part or to allay the violence of the local symptoms, and thus to aid nature.

Taken altogether it may be seen that our conception of the meaning of aiding nature differs radically from those who deem it consists in regulating the hygiene, providing a nurse, and watching the progress of events without any attempt at active intervention. Ours is not even an armed neutrality but a strong and well directed interference, taking control of the whole course of the disease and directing it in the way we wish. Taking that view, we heartily subscribe to the principle of making our one object in treating patients the aiding of nature.—American Journal of Clinical Medicine.

